# Association of systemic immune-inflammation index with severity in acute ischemic stroke patients: a cross-sectional study

**DOI:** 10.3389/fneur.2025.1553730

**Published:** 2025-06-18

**Authors:** Zichen Rao, Yiming Zhang, Chunyan Zhu

**Affiliations:** Department of Endocrinology, The Quzhou Affiliated Hospital of Wenzhou Medical University, Quzhou People’s Hospital, Quzhou, Zhejiang, China

**Keywords:** systemic immune-inflammation index (SII), acute ischemic stroke (AIS), NIHSS, inflammation, prognostic marker

## Abstract

**Objective:**

To investigate the association between the Systemic Immune-Inflammation Index (SII) and the severity of acute ischemic stroke (AIS), as measured by the National Institutes of Health Stroke Scale (NIHSS), and to explore its potential as a predictive marker for clinical outcomes.

**Methods:**

We used the data of 1723 AIS patients in the Stroke Center of Quzhou People’s Hospital from September 2016 to September 2022 for a cross-sectional study. SII was calculated as platelet count × neutrophil count divided by lymphocyte count. Stroke severity was classified as mild (NIHSS < 8) or severe (NIHSS ≥ 8). Multivariable logistic regression and subgroup analyses were performed to evaluate the relationship between SII levels and NIHSS scores, adjusting for confounders such as age, sex, and comorbidities. Nonlinear associations and threshold effects were further assessed using smooth curve fitting.

**Results:**

Elevated SII levels were independently associated with higher stroke severity (OR: 1.04, 95% CI: 1.01–1.07). A nonlinear relationship was identified, with a critical range of SII/100 values (2.4–7.8) demonstrating the strongest correlation with NIHSS scores. Patients in the highest SII quartile (Q4) exhibited a 3.46-fold increase in odds of severe stroke compared to those in the lowest quartile (Q1) (*p* < 0.001). Subgroup analyses confirmed the robustness of these findings across diverse demographic and clinical profiles.

**Conclusion:**

SII is a robust biomarker for predicting stroke severity in AIS patients. The observed nonlinear relationship highlights its potential utility in identifying critical inflammatory thresholds for risk stratification and personalized therapeutic interventions.

## Introduction

Acute ischemic stroke (AIS) is a significant cause of morbidity and mortality on a global scale. Early identification of stroke severity is of critical importance for effective clinical management and prognosis (1). The conventional approach to the evaluation of stroke severity involves the utilisation of the National Institutes of Health Stroke Scale (NIHSS), a scale designed to assess neurological function based on a range of clinical signs. However, this scale does not capture the underlying pathophysiological processes, particularly the complex immune-inflammatory responses that contribute to stroke progression and outcomes (2).

Immune-inflammatory processes contribute not only to the initial pathogenesis of AIS, but also to secondary injury through mechanisms such as microglial activation, blood–brain barrier disruption, and neuroinflammation (3, 4). These inflammatory processes are not only central to stroke pathophysiology but also serve as important biomarkers for predicting stroke severity and recovery.

The SII calculated as platelet count × neutrophil count / lymphocyte count, has emerged as a promising prognostic biomarker in various diseases, including cardiovascular conditions and cancer (5–7). By reflecting both inflammatory and thrombotic components, SII may also hold potential value in AIS.

Notwithstanding its promising applications, the role of SII in AIS remains to be thoroughly explored. Previous studies have reported that elevated SII is associated with increased stroke severity and poorer outcomes in AIS patients (8).

However, these studies have primarily focused on linear associations. To date, the potential nonlinear relationship between SII and stroke severity, as well as the existence of critical threshold effects, remains underexplored (9). To address this, we applied a generalized additive model (GAM), a flexible analytical approach that allows for the data-driven identification of complex, non-monotonic associations without imposing a prespecified functional form. Understanding these dynamics could offer significant insights into stroke prognosis and inform clinical decision-making.

In order to address this paucity of research in the field, the present study aims to investigate the association between SII and stroke severity in AIS patients. It is hypothesised that elevated SII levels are significantly associated with greater stroke severity, and that SII may serve as an independent prognostic marker for AIS outcomes. The study will provide a more in-depth understanding of the potential utility of SII in clinical practice and its role in improving the prognostication of AIS patients.

## Materials and methods

### Study population

This study utilized data from a previously established cohort examining the prognosis of cerebral infarction. Data were collected from eligible patients admitted to our hospital between September 2016 and September 2022. The study protocol was approved by the Ethics Committee of Quzhou People’s Hospital, and all participants provided written informed consent.

Inclusion criteria:

(1) Diagnosis of acute ischemic stroke (AIS) confirmed within 24 h of symptom onset.
(2) Age ≥ 18 years.
(3) Completion of head MRI within 48 h of admission.

(4) Exclusion criteria:

(1) Patients with stroke in non-acute stages or those who have transitioned to recovery phases, or with a history of brain tumors, encephalitis, traumatic brain injury, or severe multi-organ dysfunction syndrome.
(2) Pregnant patients.
(3) Severe cardiovascular conditions (NYHA class III or IV or left ventricular ejection fraction < 40%), pulmonary conditions (oxygen saturation < 95%, with shortness of breath, cyanosis, or abnormal blood gas analysis), hepatic (serum ALT > 10 times the upper reference range), renal (serum creatinine > 443 μmol/L) and oncological diseases.
(4) Patients with autoimmune diseases.
(5) Presence of infection (oral temperature > 37.5°C and white blood cell count exceeding the upper reference range).

Data collection adhered to privacy protection principles, without involving personal information, and internal data collection has not been publicly released.

### Data collection

Baseline demographic and clinical data were retrieved from medical records, including age, sex, smoking history, hypertension, type 2 diabetes, atrial fibrillation, and chronic obstructive pulmonary disease (COPD). Clinical characteristics were assessed using the NIH Stroke Scale (NIHSS) for stroke severity, the A2DS2 score (Age, Atrial fibrillation, Dysphagia, Sex, and Stroke Severity) for pneumonia risk assessment, and the Glasgow Coma Scale (GCS) for consciousness impairment.

NIHSS scores were initially assessed at the time of hospital admission by trained neurologists, and reassessed at 24 and 72 h to ensure consistency. For stroke severity, patients were classified into mild (NIHSS < 8) and severe (NIHSS ≥ 8) groups, with an NIHSS score of ≥ 8 indicating severe stroke, in accordance with previous studies that have adopted or validated this cutoff in similar AIS populations (10–12).

### Laboratory testing

Blood samples were collected by trained nurses on the second morning after admission (between 6:00 and 7:30 a.m. following overnight fasting) using vacuum tubes, stored at 4°C, and processed within 2 h by certified laboratory technicians. This standardized timing was adopted to minimize acute-phase fluctuations and nutritional influences on inflammatory markers. Laboratory tests included: White blood cell count (WBC), Neutrophil-to-lymphocyte ratio (NLR), Platelet count (P), Aspartate transaminase (AST), Alanine transaminase (ALT), Glycated hemoglobin (HbA1c), Homocysteine (HCY), Serum creatinine (Scr), Albumin (ALB), Triglycerides (TG), Total cholesterol (TC), High-density lipoprotein (HDL-c), Low-density lipoprotein (LDL-c), C-reactive protein (CRP), and uric acid (UA), all measured from the same fasting blood samples using standardized enzymatic colorimetric methods. The neutrophil-to-lymphocyte ratio was calculated as the ratio of neutrophil count to lymphocyte count. The Systemic Immune-Inflammation Index (SII) was calculated as:


SII=Platelet count×Neutrophil count/Lymphocyte count

The estimated glomerular filtration rate (eGFR) was calculated from serum creatinine using the CKD-EPI formula to assess renal function.

### Statistical analysis

All statistical analyses were performed using R Studio (version 4.2.2) and Empower Stats (version 2.0). Descriptive statistics were used to summarize baseline characteristics. Continuous variables were tested for normality using the Shapiro–Wilk test. If normally distributed, they were expressed as mean ± standard deviation and compared using t-tests. For non-normally distributed data, the Mann–Whitney U test was used, and results were presented as median and interquartile range. Categorical variables were presented as counts and percentages, and analyzed using chi-square tests.

The Systemic Immune-Inflammation Index (SII) was categorized into four quartiles (Q1 to Q4), with Q1 representing the lowest values and Q4 the highest. Stroke severity (measured by NIHSS) was compared between SII quartiles using t-tests for continuous variables and chi-square tests for categorical variables.

To further explore the relationship between SII and stroke severity, multivariate logistic regression analysis was performed, adjusting for potential confounders such as age, sex, smoking status, diabetes, hypertension, COPD, atrial fibrillation, HbA1c, eGFR, HDL-c, LDL-c, WBC, CRP, UA, and TG. Three models were constructed:

Model 1: No covariate adjustments.

Model 2: Adjusted for age and sex.

Model 3: Adjusted for both core clinical and laboratory variables. Specifically, demographic and clinical variables (age, sex, smoking status, diabetes, hypertension, COPD, and atrial fibrillation) were selected *a priori* based on their established relevance to AIS prognosis. Laboratory variables (eGFR, HDL-c, LDL-c, WBC, CRP, UA, HbA1c and TG) were included based on their statistical significance in univariate analysis (*p* < 0.10) (see Supplementary Table 1).

Additionally, a smooth curve fitting method was applied to explore the potential non-linear relationship between SII and NIHSS scores. A threshold effect analysis model based on a piecewise linear regression approach was also employed, treating NIHSS as a continuous outcome, to identify potential inflection points in the association.

Subgroup analyses were performed based on age, sex, smoking, diabetes, hypertension, atrial fibrillation, and COPD. Interaction analyses were conducted within each subgroup to assess the effects of SII. All statistical tests were two-tailed, and a significance level of *p* < 0.05 was considered statistically significant.

We did not perform *a priori* sample size calculation, as this was a retrospective analysis. However, the relatively large sample size (*N* = 1,723) provided adequate power to detect clinically relevant associations.

## Results

### Baseline characteristics of participants

There were 1723participants enrolled, of whom 40.51% were male, with an average age of 69.29 ± 12.42 years. The mean SII median (IQR) concentrations were 378.13 (252.15–572.11). Among them, severe stroke patients accounted for16.42%.

The clinical characteristics of participants with stroke severity as a columnar stratified variable are shown in Table 1. Patients with NIHSS ≥ 8 were significantly older, had higher prevalence of atrial fibrillation (31.8% vs. 13.2%) and COPD (13.43% vs. 5.00%), and exhibited elevated inflammatory markers (SII: severe group 533.21 vs. mild group 359.90, *p* < 0.001; CRP: severe group 6.55 vs. mild group 2.26, *p* < 0.001). These patients also showed worse clinical scores (e.g., GCS: severe group 12 vs. mild group 15, *p* < 0.001). This highlights the association between systemic inflammation and stroke severity, alongside comorbidities like atrial fibrillation and COPD.

**Table 1:** Baseline characteristics of patients stratified by NIHSS (< 8 vs. ≥ 8).

Characteristics	NIHSS < 8 (*N* = 1,440)	NIHSS ≥ 8 (*N* = 283)	*p*-value*
Demographic characteristic			
Age years, mean ± SD	68.74 ± 12.28	72.14 ± 12.81	< 0.001
Sex (%)			0.007
Male	563 (39.10%)	135 (47.70%)	
Female	877 (60.90%)	148 (52.30%)	
Current smoking, *n* (%)			0.859
No	913 (63.40%)	181 (63.96%)	
Yes	527 (36.60%)	102 (36.04%)	
Hypertension, *n* (%)			0.414
No	329 (22.85%)	71 (25.09%)	
Yes	1,111 (77.15%)	212 (74.91%)	
Diabetes, *n* (%)			0.106
No	920 (63.89%)	195 (68.90%)	
Yes	520 (36.11%)	88 (31.10%)	
Atrial fibrillation, *n* (%)			< 0.001
No	1,250 (86.81%)	193 (68.20%)	
Yes	190 (13.19%)	90 (31.80%)	
COPD, *n* (%)			< 0.001
No	1,368 (95.00%)	245 (86.57%)	
Yes	72 (5.00%)	38 (13.43%)	
Laboratory parameters			
HbA1c, (%), median (IQR)	6.00 (5.60–7.20)	6.00 (5.50–7.20)	0.676
TG, nmol/L, mean ± SD	1.57 ± 1.05	1.27 ± 0.72	< 0.001
Albumin, g/L, mean ± SD	38.11 ± 3.74	37.17 ± 4.40	< 0.001
LDL-C, mmol/L, mean ± SD	2.86 ± 1.01	2.83 ± 0.93	0.869
HDL-C, mmol/L, mean ± SD	1.16 ± 0.31	1.19 ± 0.30	0.135
Homocysteine, mmol/L, median (IQR)	14.90(11.70–19.06)	15.00 (11.90–19.95)	0.323
CRP, mg/L, median (IQR)	2.26 (1.00–5.00)	6.55 (2.54–18.14)	< 0.001
WBC, ×10^9^/L, mean ± SD	7.20 ± 2.56	8.26 ± 3.05	< 0.001
EGFR, ml/min/1.73m^2^, mean ± SD	101.50 ± 33.99	96.86 ± 32.64	0.067
UA, umol/L, mean ± SD	324.74 ± 96.99	306.73 ± 102.10	0.011
SII, ×10^9^/L, median (IQR)	359.90 (248.09–526.53)	533.21 (307.84–829.12)	< 0.001
Clinical characteristics			
A2DS2, median (IQR)	2.00 (2.00–3.00)	6.00 (4.00–7.00)	< 0.001
GCS, median (IQR)	15.00(15.00–15.00)	12.00 (10.00–13.00)	< 0.001
NIHSS, median (IQR)	2.00 (1.00–4.00)	12.00 (9.00–16.00)	< 0.001

NIHSS, National Institutes of Health Stroke Scale; Sex, biological sex (male/female); COPD, chronic obstructive pulmonary disease; IQR, interquartile range; HbA1c, Hemoglobin A1c; TG, triglycerides; LDL-C, low-density lipoprotein cholesterol; HDL-C, high-density lipoprotein cholesterol; CRP, C-reactive protein; WBC, white blood cell; eGFR, estimated glomerular filtration rate; UA, uric acid; SII, systemic immune-inflammation index; A2DS2, Age, Atrial fibrillation, Dysphagia, Sex, and Stroke Severity score; GCS, Glasgow Coma Scale. *p < 0.05 was considered statistically significant.

The clinical characteristics of the participants according to the quartiles of SII are shown in Table 2. In this study, significant differences in patient characteristics were observed across SII quartiles. Patients in the highest quartile (Q4) were more likely to have atrial fibrillation (23.20% vs. 13.23%, *p* < 0.001) and COPD (9.28% vs. 5.57%, *p* = 0.032) compared to the lowest quartile (Q1). Inflammatory markers, including CRP (5.71 mg/L vs. 2.00 mg/L, *p* < 0.001) and WBC (9.39 × 10⁹/L vs. 6.13 × 10⁹/L, *p* < 0.001), increased significantly with higher SII levels. Conversely, triglyceride levels decreased across quartiles (1.33 vs. 1.56 mmol/L, *p* < 0.001).

**Table 2:** Comparison of patient characteristics across SII quartiles.

Characteristics	Q1 (*N* = 431)	Q2 (*N* = 430)	Q3 (*N* = 431)	Q4 (*N* = 431)	*p*-value*
Demographic characteristic					
Age years, mean ± SD	69.60 ± 11.57	68.44 ± 12.94	69.13 ± 12.58	70.02 ± 12.56	0.254
Sex (%)					0.023
Male	165 (38.28%)	157 (36.51%)	177 (41.07%)	199 (46.17%)	
Female	266 (61.72%)	273 (63.49%)	254 (58.93%)	232 (53.83%)	
Current smoking, *n* (%)					0.168
No	267 (61.95%)	259 (60.23%)	279 (64.73%)	289 (67.05%)	
Yes	164 (38.05%)	171 (39.77%)	152 (35.27%)	142 (32.95%)	
Hypertension, *n* (%)					0.120
No	117 (27.15%)	96 (22.33%)	99 (22.97%)	88 (20.42%)	
Yes	314 (72.85%)	334 (77.67%)	332 (77.03%)	343 (79.58%)	
Diabetes, *n* (%)					0.192
No	264 (61.25%)	281 (65.35%)	276 (64.04%)	294 (68.21%)	
Yes	167 (38.75%)	149 (34.65%)	155 (35.96%)	137 (31.79%)	
Atrial fibrillation, *n* (%)					<0.001
No	374 (86.77%)	375 (87.21%)	363 (84.22%)	331 (76.80%)	
Yes	57 (13.23%)	55 (12.79%)	68 (15.78%)	100 (23.20%)	
COPD, *n* (%)					0.032
No	407 (94.43%)	404 (93.95%)	411 (95.36%)	391 (90.72%)	
Yes	24 (5.57%)	26 (6.05%)	20 (4.64%)	40 (9.28%)	
Laboratory parameters					
A1c, (%), median (IQR)	6.10 (5.60–7.20)	5.90 (5.50–7.20)	6.10 (5.50–7.20)	6.00 (5.60–7.00)	0.419
TG, nmol/L, mean ± SD	1.56 ± 1.10	1.67 ± 1.13	1.52 ± 0.96	1.33 ± 0.78	< 0.001
LDL-C, mmol/L, mean ± SD	2.74 ± 0.89	2.80 ± 0.98	2.93 ± 1.04	2.96 ± 1.06	0.004
HDL-C, mmol/L, mean ± SD	1.17 ± 0.31	1.13 ± 0.29	1.15 ± 0.34	1.21 ± 0.30	< 0.001
Homocysteine, mmol/L, median (IQR)	14.40 (11.55–19.10)	14.30 (11.49–19.00)	15.20 (11.90–19.40)	15.70 (12.50–19.40)	0.017
CRP, mg/L, median (IQR)	2.00 (1.00–4.00)	2.00 (0.96–4.00)	3.00 (1.21–6.41)	5.71 (2.30–17.80)	< 0.001
WBC, ×10^9^/L, mean ± SD	6.13 ± 2.11	6.63 ± 1.90	7.35 ± 2.03	9.39 ± 3.22	< 0.001
EGFR, ml/min/1.73m^2^, mean ± SD	102.69 ± 32.07	98.03 ± 32.27	100.16 ± 31.73	102.05 ± 38.60	0.205
UA, umol/L, mean ± SD	326.80 ± 99.03	330.79 ± 97.49	317.95 ± 88.54	311.59 ± 105.56	0.014
SII, ×10^9^/L, median (IQR)	199.29 (152.75–225.12)	313.52 (282.00–343.34)	461.45 (421.11–506.37)	810.70 (660.13–1063.38)	< 0.001
Clinical characteristics					
A2DS2, median (IQR)	2.00 (2.00–4.00)	2.00 (2.00–4.00)	2.00 (2.00–4.00)	3.00 (2.00–5.00)	< 0.001
GCS, median (IQR)	15.00 (15.00–15.00)	15.00 (15.00–15.00)	15.00 (14.00–15.00)	15.00 (13.00–15.00)	< 0.001
NIHSS, median (IQR)	2.00 (1.00–4.00)	2.00 (1.00–4.75)	3.00 (1.00–5.00)	4.00 (2.00–8.50)	< 0.001

SII, systemic immune-inflammation index; Sex, biological sex (male/female); COPD, chronic obstructive pulmonary disease; IQR, interquartile range; TG, triglycerides; LDL-C, low-density lipoprotein cholesterol; HDL-C, high-density lipoprotein cholesterol; CRP, C-reactive protein; WBC, white blood cell; eGFR, estimated glomerular filtration rate; UA, uric acid; A2DS2, Age, Atrial fibrillation, Dysphagia, Sex, and Stroke Severity score; GCS, Glasgow Coma Scale. *p < 0.05 was considered statistically significant.

Stroke severity, as assessed by NIHSS scores, increased across SII quartiles, with the median NIHSS rising from 2 in Q1 to 4 in Q4 (*p* < 0.001). GCS and A2DS2 scores also differed significantly across quartiles, with lower GCS and higher A2DS2 scores observed in patients with higher SII levels (all *p* < 0.001).

### Association between SII and NIHSS

To improve interpretability and model scaling, the SII was divided by 100 before inclusion in regression analyses. Table 3 presents the results of the multivariable regression analysis between SII/100 and NIHSS. This association, modeled as a continuous variable (per 100-unit increase in SII), was significant in all three models: model 1 (OR = 1.09, 95% CI: 1.06–1.12), model 2 (OR = 1.09, 95% CI: 1.06–1.11), and model 3 (OR = 1.04, 95% CI: 1.01–1.07). Although statistically significant, the effect size was modest, suggesting limited predictive power of SII when treated as a continuous variable. In the fully adjusted model, SII in the highest quartile (Q4) was associated with 3.46-fold higher odds of severe stroke (NIHSS ≥ 8) compared to the lowest quartile (*p* < 0.001). These results confirm that elevated SII independently correlates with greater stroke severity, even after controlling for age, comorbidities, and other inflammatory markers.

**Table 3:** Multivariable regression models for SII predicting NIHSS severity.

	Crude model (Model 1)	Partially adjusted model (Model 2)	Fully adjusted model (Model 3)
	*β* (95% CI) *p*-value	*β* (95% CI) *p*-value	*β* (95% CI) *p*-value
SII/100	1.09 (1.06, 1.12)*	1.09 (1.06, 1.11)*	1.04 (1.01, 1.07)*
SII quartiles			
Quartile 1	Reference	Reference	Reference
Quartile 2	0.96 (0.63, 1.46)	0.98 (0.64, 1.49)	0.97 (0.64, 1.49)
Quartile 3	1.33 (0.89, 1.97)	1.33 (0.90, 1.98)	1.34 (0.90, 2.00)
Quartile 4	3.48 (2.44, 4.98)*	3.44 (2.40, 4.93)*	3.46 (2.41, 4.97)*
*p* for trend	< 0.001	< 0.001	0.0195

Model 1, no covariates were adjusted. Model 2, age, sex were adjusted. Model 3, age, sex, Current smoking, hypertension, diabetes, COPD, Atrial fibrillation, HDL-C, LDL-C, WBC, CRP, EGFR, UA, A1c and TG were adjusted. 95% CI, 95% confidence interval; OR, odds ratio; SII, systemic immunity-inflammation index. * *p* < 0.001 was considered statistically significant.

**Figure 1:**
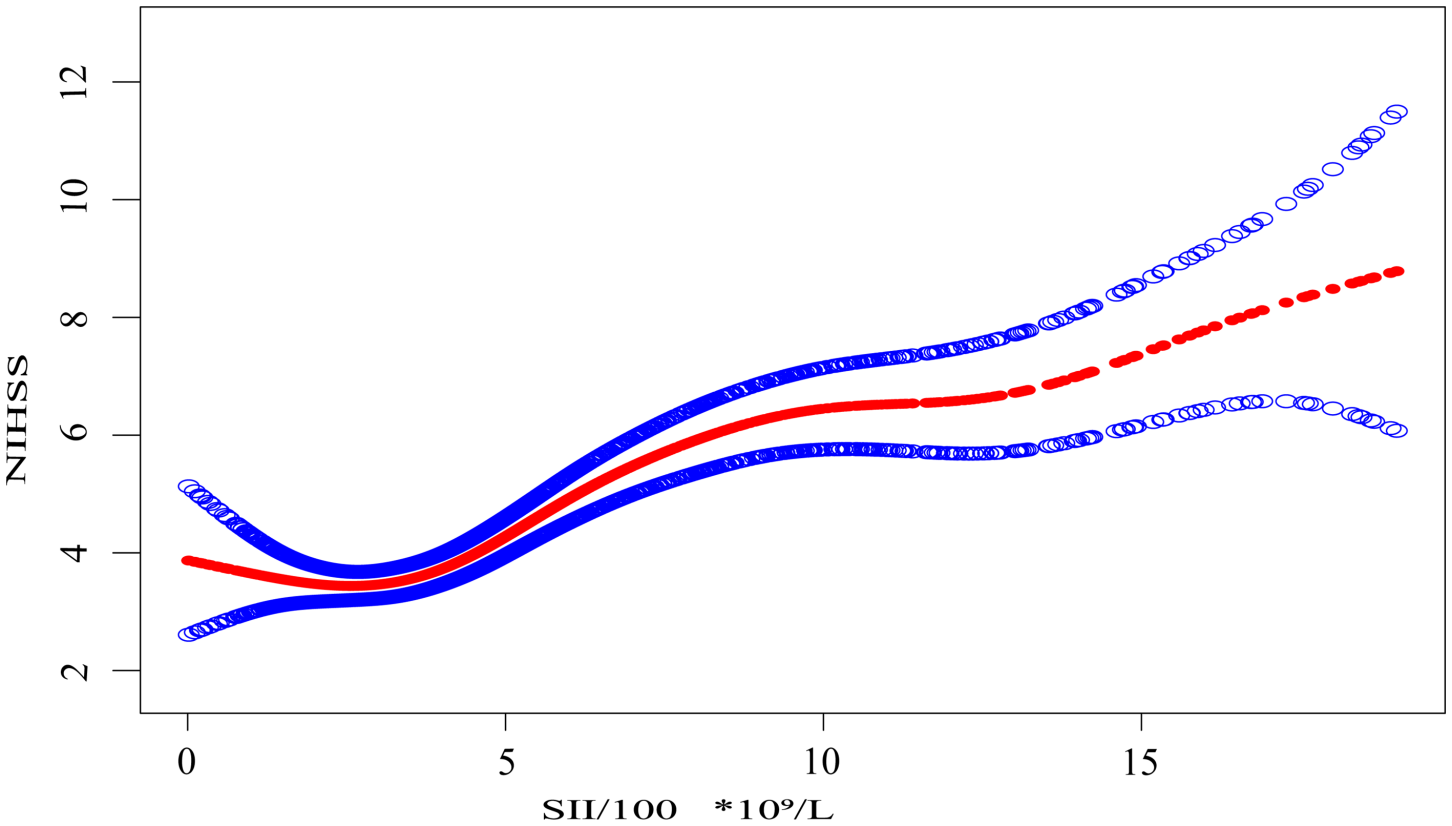
The association between the systemic immune-inflammation index scaled by 100 (SII/100, ×10⁹/L) and NIHSS score. The solid red line represents the smooth curve fit between the scaled SII and NIHSS. Shaded blue bands indicate the 95% confidence interval of the fit. SII was divided by 100 to facilitate interpretation of effect sizes in regression modeling.

Based on model 3, which adjusted for potential confounders including age, sex, smoking, diabetes, hypertension, COPD, atrial fibrillation, HbA1c, eGFR, HDL-c, LDL-c, WBC, CRP, UA, and TG, we performed smooth curve fitting and threshold effect analysis to examine the relationship between SII and NIHSS. The results are presented in Figure 1, Tables 4, 5.

**Table 4:** Threshold effect analysis of SII on NIHSS at the inflection point of 2.4 using a two-piecewise linear regression model.

NIHSS	Adjust *β* (95% CI)	*p*
SII/100		
Fitting by standard linear model	0.3 (0.3, 0.4)	< 0.001
Fitting by two-piecewise linear model		
Inflection point	2.4	
< 2.4	−0.5 (−1.2, 0.2)	0.147
> 2.4	0.4 (0.3, 0.5)	< 0.001
Log-likelihood ratio	0.014	

Age, sex, current smoking, hypertension, diabetes, COPD, Atrial fibrillation, HDL-C, LDL-C, WBC, CRP, EGFR, UA, A1c and TG were adjusted.

**Table 5:** Threshold effect analysis of SII on NIHSS at the inflection point of 7.8 using a two-piecewise linear regression model.

NIHSS	Adjust *β* (95% CI)	*p*
SII/100		
Fitting by standard linear model	0.4 (0.3, 0.5)	< 0.001
Fitting by two-piecewise linear model		
Inflection point	7.8	
< 7.8	0.5 (0.4, 0.7)	< 0.001
> 7.8	0.2 (0.0, 0.4)	0.022
Log-likelihood ratio	0.035	

Age, sex, current smoking, hypertension, diabetes, COPD, Atrial fibrillation, HDL-C, LDL-C, WBC, CRP, EGFR, UA, A1c and TG were adjusted.

Smooth curve fitting, as shown in Figure 1, provides an intuitive visualization of the nonlinear relationship between SII/100 and NIHSS. The curve shows an initial flat segment, followed by an upward trend, and finally a plateauing tendency at higher SII/100 values. While the smooth curve fit suggests a nonlinear relationship, it does not allow for the precise identification of inflection points. To further quantify this relationship and identify key thresholds, we performed a threshold effect analysis using a two-piece linear regression model.

Threshold effect analysis identified two statistically significant inflection points at SII/100 values of 2.4 and 7.8. When modeled continuously, NIHSS increased by approximately 0.4–0.5 points per 100-unit increase in SII within the midrange (SII/100 = 2.4–7.8), but this association flattened beyond 7.8. Specifically, when SII/100 was below 2.4, the association was not statistically significant (adjusted *β* = −0.5, 95% CI: −1.2 to 0.2, *p* = 0.147). Between 2.4 and 7.8, a strong positive association was observed, with β increasing from 0.4 (95% CI: 0.3–0.5, *p* < 0.001) to 0.5 (95% CI: 0.4 to 0.7, *p* < 0.001). When SII/100 exceeded 7.8, the association was attenuated but remained statistically significant (adjusted *β* = 0.2, 95% CI: 0.0 to 0.4, *p* = 0.022). These values represent adjusted regression coefficients (β), indicating changes in NIHSS score per 100-unit increase in SII, not odds ratios.

A log-likelihood ratio test confirmed that the two-piecewise linear regression model provided a significantly better fit than a standard linear model (*p* < 0.05), supporting the presence of threshold effects without overcomplicating interpretation.

Further subgroup analysis showed that SII consistently predicted the severity of NIHSS, as shown in Table 6. No significant interactions were detected between SII and subgroups such as age, sex, hypertension, or atrial fibrillation (all P for interaction > 0.1). This supports the robustness of SII as a biomarker across diverse patient characteristics. However, the effect sizes in each subgroup were relatively small, with *β* values typically ranging from 0.0 to 0.1.

**Table 6:** Subgroup analysis for SII’s predictive effect across patient characteristics.

Exposure	OR, 95%CI	*p*-value	P for interaction
Sex			0.1014
Male	0.1 (0.0, 0.2)	0.0050	
Female	0.0 (0.0, 0.1)	0.0008	
Age			0.1953
≤75	0.0 (0.0, 0.1)	0.0003	
>75	0.1 (0.0, 0.2)	0.0176	
Hypertension			0.0524
No	0.0 (0.0, 0.1)	0.0041	
Yes	0.1 (0.0, 0.2)	0.0009	
Diabetes			0.7287
No	0.1 (0.0, 0.1)	0.0001	
Yes	0.1 (−0.0, 0.1)	0.1044	
Atrial fibrillation			0.6085
No	0.0 (0.0, 0.1)	0.0002	
Yes	0.1 (−0.0, 0.2)	0.2028	
Current smoking			0.8027
No	0.1 (0.0, 0.1)	<0.0001	
Yes	0.0 (−0.1, 0.1)	0.4076	
COPD			0.1174
No	0.1 (0.0, 0.1)	<0.0001	
Yes	−0.1 (−0.4, 0.1)	0.2597	

OR, odds ratio; 95% CI, 95% confidence interval; COPD, chronic obstructive pulmonary disease.

To enhance interpretability and address clinical relevance, we additionally performed subgroup analyses using logistic regression with NIHSS ≥ 8 as the outcome. These results, presented in Supplementary Table 1, demonstrate that SII remained a consistent predictor of severe stroke across all subgroups, with no significant effect modification detected.

## Discussion

In this cross-sectional study, a significant association was identified between the Systemic Immune-Inflammation Index (SII) and stroke severity, as measured by the NIHSS score. Higher SII levels were consistently associated with more severe neurological deficits, even after adjusting for key demographic and clinical factors. Of particular note was the observation of a non-linear relationship, with critical thresholds identified at SII/100 values of 2.4 and 7.8, suggesting that the impact of SII on stroke severity varies across its range. Subgroup analyses further confirmed the robustness of this association across different patient characteristics, including age, sex, and comorbid conditions. These findings underscore the potential of SII as a reliable and easily accessible biomarker for assessing stroke severity upon admission.

In addition to elevated inflammatory markers, patients with severe stroke showed lower levels of triglycerides and serum albumin. This pattern may reflect metabolic stress or subclinical malnutrition, which are common in acute stroke. The reduction in albumin further supports a catabolic state, often associated with worse prognosis. These findings underscore the relevance of nutritional and metabolic markers in assessing stroke severity.

The role of inflammation in acute ischemic stroke (AIS) has become increasingly recognised, with mounting evidence associating elevated inflammatory biomarkers—such as C-reactive protein (CRP), interleukins (e.g., IL-6), and tumor necrosis factor-alpha (TNF-*α*)-with more severe strokes and poorer functional recovery (13, 14). Furthermore, studies have identified the Systemic Immune-Inflammation Index (SII) as a potential marker for stroke severity and prognosis (7). For instance, Hou et al. (15) emphasised the predictive value of SII in distinguishing between severe and mild strokes. A previous study by Huang et al. (8) also demonstrated that elevated SII is associated with increased stroke severity and poor functional outcomes in AIS patients. However, unlike our work, their analysis did not explore nonlinear effects or threshold inflection points. The present study corroborates these findings by demonstrating a significant correlation between SII and NIHSS, thereby highlighting systemic inflammation’s central role in AIS pathophysiology. Elevated SII has been shown to reflect a heightened thrombosis state (increased platelet count), immune imbalance (elevated neutrophil count), and impaired adaptive immunity (reduced lymphocyte count) (7). Collectively, these factors contribute to increased ischemic brain injury through neuroinflammation, endothelial dysfunction, and blood–brain barrier disruption. Elevated SII reflects an imbalance in key immune-inflammatory components-neutrophils, platelets, and lymphocytes-that jointly mediate secondary brain injury in acute ischemic stroke. Neutrophils are among the earliest immune cells recruited to ischemic brain tissue, where they contribute to blood–brain barrier disruption, generate reactive oxygen species, and form neutrophil extracellular traps (NETs), all of which amplify neuroinflammation and exacerbate tissue damage (16). Platelets, in addition to their pro-thrombotic role, promote endothelial activation and leukocyte adhesion, facilitating microvascular occlusion and further ischemia in the penumbral region (17). Concurrently, lymphopenia, particularly reduced regulatory T cells, impairs immune homeostasis, diminishes anti-inflammatory regulation, and has been associated with worse functional outcomes in AIS (18). SII, as a composite index, captures this inflammatory triad-excessive neutrophilic and platelet activity combined with suppressed lymphocyte-mediated regulation-offering a more integrated representation of the systemic immune-inflammatory state than any single component alone. These mechanisms not only explain the observed association between SII and stroke severity, but also underscore the potential clinical value of targeting these pathways to mitigate secondary injury and improve neurological recovery. The exacerbation of tissue damage and acceleration of neuronal death is further compounded by the activation of immune cells, particularly the recruitment of neutrophils and macrophages to the ischemic site (19, 20). While inflammation undoubtedly contributes to the acute phase of stroke, emerging studies (21–23) suggest that early anti-inflammatory interventions may offer significant therapeutic benefits. Clinical trials employing anti-inflammatory agents, including corticosteroids, interleukin inhibitors, and other immunomodulatory drugs, have demonstrated encouraging results in mitigating post-stroke neuroinflammation and enhancing functional recovery. Nevertheless, challenges persist in identifying patients who would benefit most from such therapies and in balancing the risks of immune suppression with the benefits of controlling inflammation.

SII is a composite biomarker that integrates three critical components of the systemic inflammatory and thrombotic response: platelets, neutrophils, and lymphocytes (24). Unlike the neutrophil-to-lymphocyte ratio (NLR), which reflects only the balance between innate and adaptive immune cells, SII additionally incorporates platelet count, capturing the prothrombotic and microvascular contributions to stroke pathology (25). This may offer a more comprehensive representation of the inflammatory state in AIS. Furthermore, while cytokines such as interleukin-6 (IL-6) have been linked to stroke severity and outcomes (26), they are less accessible in routine clinical settings due to higher cost, longer turnaround time, and variability in measurement techniques. By contrast, SII is derived from standard complete blood counts, making it a cost-effective and easily obtainable marker with practical prognostic value.

The present study identifies a key ‘inflammatory tipping point’ between SII/100 values of 2.4 and 7.8, which may suggest that moderate levels of systemic inflammation are particularly associated with worse neurological deficits in AIS patients. When SII is low, it generally reflects a higher lymphocyte-to-neutrophil and platelet ratio, possibly indicating a less activated immune-inflammatory state. In such instances, the immune response may have limited capacity to influence stroke outcomes, and the presence of immune tolerance mechanisms could theoretically contribute to the modulation of ischemic injury (27), although this interpretation remains speculative and unsupported by direct evidence in our study. This may provide a rationale for the observation that patients with low SII tend to experience less severe strokes. Conversely, when SII is elevated, indicating heightened immune activation, the immune system might reach a saturation threshold beyond which additional activation may not further worsen ischemic damage. Moreover, elevated levels of systemic inflammation may potentially trigger immune dysregulation and activate compensatory anti-inflammatory pathways (28), which could help limit additional neurological deterioration, though this remains a hypothesis. Despite robust immune activation, excessive inflammation may not amplify ischemic damage, as the immune system’s self-regulatory mechanisms help constrain further injury (29). This proposed immunological feedback may partially explain the attenuated correlation observed between higher SII values and stroke severity, but further research is needed to confirm this mechanism (30).

To our knowledge, this study is one of the first to systematically examine the nonlinear association between SII and NIHSS in AIS patients, using smooth curve modeling and threshold effect analysis (31). The statistical adjustments and subgroup analyses employed in this study enhance the reliability and generalizability of the findings. However, as a single-center, cross-sectional study, it is not possible to infer causality, and the findings may not be fully generalizable to other populations. Regional differences in patient demographics, stroke subtypes, and institutional treatment protocols may influence baseline inflammatory levels and, consequently, the observed threshold effects of SII. Validation across diverse clinical settings is therefore necessary to confirm the broader applicability of these findings. While we adjusted for a wide range of potential confounders, the possibility of residual confounding from unmeasured variables cannot be excluded. Additionally, the cross-sectional design limits our ability to determine the temporal sequence between elevated SII and stroke severity. Additionally, since SII was measured on the second day of hospitalization rather than upon admission, it may partially reflect the early inflammatory response to stroke severity rather than purely predict it. Despite implementing extensive confounder adjustment, the potential for residual confounding from unmeasured variables remains unaccounted for. Future research should focus on the temporal dynamics of SII during the acute and recovery phases of AIS to determine its potential as a long-term prognostic marker. Hence, larger, multicentre, longitudinal studies are needed to further explore whether modulating SII levels could improve outcomes and reduce the burden of disability in AIS patients.

## Conclusion

This study suggests that the SII is independently associated with stroke severity in patients with AIS, and that this association may be nonlinear, with critical threshold effects observed. SII may hold potential as a biomarker for stratifying stroke severity, but its clinical utility requires further validation. Future longitudinal and interventional studies are warranted to confirm these findings, elucidate causal pathways, and determine whether modulating systemic inflammation could improve clinical outcomes in AIS.

## Data Availability

The raw data supporting the conclusions of this article will be made available by the authors, without undue reservation.
