# Supplementary material for: Association of systemic immune-inflammation index with severity in acute ischemic stroke patients: a cross-sectional study
Source: Front Neurol. 2025 Jun 18;16:1553730. doi: 10.3389/fneur.2025.1553730 (PMC12213794; doi:10.3389/fneur.2025.1553730)
Supplement: Supplementary file 1 [file Table_1.docx]

**Supplementary Table 1. Univariable Logistic Regression Analysis of Baseline Characteristics Associated with Severe Stroke (NIHSS ≥ 8)**

| Variable | Statistics | OR (95% CI) | P-value |
| --- | --- | --- | --- |
| Age years, n (%) |  |  |  |
| >75 | 587 (34.07%) | Referece |  |
| <=75 | 1136 (65.93%) | 0.65 (0.50, 0.85) | 0.0013 |
| Sex |  |  |  |
| Male | 698 (40.51%) | Referece |  |
| Female | 1025 (59.49%) | 0.70 (0.54, 0.91) | 0.0072 |
| Current smoking, n (%) |  |  |  |
| No | 1094 (63.49%) | Referece |  |
| Yes | 629 (36.51%) | 0.98 (0.75, 1.27) | 0.8593 |
| Hypertension, n (%) |  |  |  |
| No | 400 (23.22%) | Referece |  |
| Yes | 1323 (76.78%) | 0.88 (0.66, 1.19) | 0.4145 |
| Diabetes, n (%) |  |  |  |
| No | 1115 (64.71%) | Referece |  |
| Yes | 608 (35.29%) | 0.80 (0.61, 1.05) | 0.1070 |
| Atrial fibrillation, n (%) |  |  |  |
| No | 1443 (83.75%) | Referece |  |
| Yes | 280 (16.25%) | 3.07 (2.29, 4.11) | <0.0001 |
| COPD |  |  |  |
| No | 1613 (93.62%) | Referece | <0.0001 |
| Yes | 110 (6.38%) | 2.95 (1.94, 4.47) |  |
| HbA1c (%)，mean ± SD | 6.63 ± 1.71 | 1.00 (0.92, 1.09) | 0.9376 |
| TG (nmol/L), mean ± SD | 1.52 ± 1.01 | 0.63 (0.52, 0.77) | <0.0001 |
| HDL-C (mmol/L), mean ± SD | 1.17 ± 0.31 | 1.27 (0.86, 1.88) | 0.2379 |
| LDL-C (mmol/L), mean ± SD | 2.85 ± 1.00 | 0.97 (0.86, 1.11) | 0.6915 |
| UA (umol/L), mean ± SD | 321.78 ± 98.05 | 1.00 (1.00, 1.00) | 0.0048 |
| CRP (mg/L), mean ± SD | 9.85 ± 26.47 | 1.02 (1.01, 1.02) | <0.0001 |
| WBC (×109/L), mean ± SD | 7.38 ± 2.68 | 1.14 (1.09, 1.19) | <0.0001 |
| EGFR (ml/min/1.73m^2^), mean ± SD | 100.73 ± 33.81 | 1.00 (0.99, 1.00) | 0.0349 |

NIHSS, National Institutes of Health Stroke Scale; Sex, biological sex (male/female); COPD, chronic obstructive pulmonary disease; HbA1c, Hemoglobin A1c; TG, triglycerides; LDL-C, low-density lipoprotein cholesterol; HDL-C, high-density lipoprotein cholesterol; UA, uric acid; CRP, C-reactive protein; WBC, white blood cell; eGFR, estimated glomerular filtration rate;

OR, odds ratio; 95% CI, 95% confidence interval; COPD, chronic obstructive pulmonary disease.
