# Supplementary material for: Association of systemic immune-inflammation index with severity in acute ischemic stroke patients: a cross-sectional study
Source: Front Neurol. 2025 Jun 18;16:1553730. doi: 10.3389/fneur.2025.1553730 (PMC12213794; doi:10.3389/fneur.2025.1553730)
Supplement: Supplementary file 2 [file Table_2.docx]

**Supplementary Table 2. Subgroup Analysis for SII’s Predictive Effect Across Patient Characteristics**

| Exposure | OR, 95%CI | p-value | P for interaction |
| --- | --- | --- | --- |
| Age |  |  | 0.54 |
| ≤ 75 | 1.11 (1.03, 1.19) | 0.0067 |  |
| ＞75 | 1.06 (0.98, 1.15) | 0.1160 |  |
| Sex |  |  | 0.33 |
| Male | 1.05 (0.98, 1.13) | 0.1752 |  |
| Female | 1.11 (1.03, 1.20) | 0.0067 |  |
| Hypertension |  |  | 0.43 |
| No | 1.06 (0.94, 1.20) | 0.3707 |  |
| Yes | 1.10 (1.04, 1.17) | 0.0008 |  |
| Diabetes |  |  | 0.96 |
| No | 1.09 (1.02, 1.17) | 0.0104 |  |
| Yes | 1.07 (0.98, 1.16) | 0.1291 |  |
| Atrial fibrillation |  |  | 0.51 |
| No | 1.12 (1.05, 1.20) | 0.0003 |  |
| Yes | 0.95 (0.87, 1.05) | 0.3322 |  |
| Current smoking |  |  | 0.49 |
| No | 1.10 (1.03, 1.17) | 0.0044 |  |
| Yes | 1.06 (0.97, 1.17) | 0.1896 |  |
| COPD |  |  | 0.54 |
| No | 1.09 (1.03, 1.15) | 0.0019 |  |
| Yes | 0.97 (0.78, 1.19) | 0.7450 |  |

SII, Systemic Immune-Inflammation Index; COPD, chronic obstructive pulmonary disease;

OR, odds ratio; 95% CI, 95% confidence interval; COPD, chronic obstructive pulmonary disease.
